# Capsular Types of *Klebsiella pneumoniae* Revisited by *wzc* Sequencing

**DOI:** 10.1371/journal.pone.0080670

**Published:** 2013-12-09

**Authors:** Yi-Jiun Pan, Tzu-Lung Lin, Yen-Hua Chen, Chun-Ru Hsu, Pei-Fang Hsieh, Meng-Chuan Wu, Jin-Town Wang

**Affiliations:** 1 Department of Microbiology, National Taiwan University College of Medicine, Taipei, Taiwan; 2 Department of Internal Medicine, National Taiwan University Hospital, Taipei, Taiwan; Université d'Auvergne Clermont 1, France

## Abstract

Capsule is an important virulence factor in bacteria. A total of 78 capsular types have been identified in *Klebsiella pneumoniae*. However, there are limitations in current typing methods. We report here the development of a new genotyping method based on amplification of the variable regions of the *wzc* gene. Fragments corresponding to the variable region of *wzc* were amplified and sequenced from 76 documented capsular types of reference or clinical strains. The remaining two capsular types (reference strains K15 and K50) lacked amplifiable *wzc* genes and were proven to be acapsular. Strains with the same capsular type exhibited ≧94% DNA sequence identity across the variable region (CD1-VR2-CD2) of *wzc*. Strains with distinct K types exhibited <80% DNA sequence identity across this region, with the exception of three pairs of strains: K22/K37, K9/K45, and K52/K79. Strains K22 and K37 shared identical capsular polysaccharide synthesis (*cps*) genes except for one gene with a difference at a single base which resulted in frameshift mutation. The *wzc* sequences of K9 and K45 exhibited high DNA sequence similarity but possessed different genes in their *cps* clusters. K52 and K79 exhibited 89% *wzc* DNA sequence identity but were readily distinguished from each other at the DNA level; in contrast, strains with the same capsular type as K52 exhibited 100% *wzc* sequence identity. A total of 29 strains from patients with bacteremia were typed by the *wzc* system. *wzc* DNA sequences confirmed the documented capsular type for twenty-eight of these clinical isolates; the remaining strain likely represents a new capsular type. Thus, the *wzc* genotyping system is a simple and useful method for capsular typing of *K. pneumoniae*.

## Introduction

*Klebsiella pneumoniae* is an important human pathogen in both hospital and community settings. This species causes nosocomial infections, such as septicemia, pneumonia, urinary tract infections, surgical site infections and catheter-related infections [1], [2], and is also associated with community-acquired infections, such as pyogenic liver abscess (PLA) complicated with meningitis and endophthalmitis, soft tissue abscesses, urinary tract infections, and pneumonia [3]–[11]. Community-acquired PLA caused by *K. pneumoniae* with or without meningitis and endophthalmitis metastatic complications represents an emerging infectious disease worldwide [6], [9], [10], [12]–[16]. Approximately 50% of patients with community-acquired PLA *K. pneumoniae* infections exhibit no apparent underlying disease, whereas the remainder of this population harbors predisposing conditions such as diabetes mellitus [13].

Capsule is a major virulence factor of *K. pneumoniae*, and capsular types are related to the severity of infection [17], [18]. The prevalence of capsular types in each *K. pneumoniae*-related disease could be crucial for disease control and prevention. However, determination of capsular types often is difficult due to the limitations of traditional serotyping [19], [20]. The results of serotyping also are inconsistent, except in patients with community-acquired PLA [8], [13], [19], [21]–[24].

Molecular methods based on the capsule polysaccharide synthesis (*cps*) region have been developed for *K. pneumoniae* capsular typing. For example, polymerase chain reaction-based genotyping of the capsular polysaccharide synthesis region, *cps* (*wzy*)-PCR genotyping, was first adopted for *K. pneumoniae* type K1 [6], [7], [25]–[28], and subsequently applied for other capsular types related to community-acquired PLA [19], [29], [30]. However, only capsular types with known sequences of capsule specific genes (e.g., *wzy*) can be typed, and a separate pair of primers is needed for each type. PCR amplification of the *cps* gene cluster (∼20 kb) followed by restriction enzyme digestion, i.e., *cps* PCR-restriction fragment length polymorphism (RFLP) analysis, is another commonly used method. Capsular types can be distinguished based on distinct RFLP profiles (C-patterns) [31]; however, amplifications of the *cps* region can be very difficult in some strains. In addition, different C-patterns have been observed in some strains that share same capsular type.

As described here, we have developed a new method for capsular typing of *K. pneumoniae* based on the sequence of the variable region of a gene, *wzc*, that encodes a capsule synthesis-related tyrosine kinase.

## Materials and Methods

### Ethics statement

The clinical strains used in this study were provided from the strain collection of National Taiwan University Hospital, En Chu Kong Hospital, Far Eastern Memorial Hospital, Chang Gung Memorial Hospital in Taiwan. The Ethics Committee confirmed that no formal ethical approval was needed to use these clinically obtained materials, because the strains were remnants from patient samples, and the data were analyzed anonymously.

### Bacterial strains

A total of 77 K-serotype *Klebsiella* reference strains purchased from Statens Serum Institute, Copenhagen, Denmark. An additional strain (A1517) of novel type KN1 was identified in a previous study from our laboratory [19]. Another eleven *K. pneumoniae* clinical isolates were obtained from Taiwanese and overseas clinical laboratories, including National Taiwan University Hospital (NTUH; Taipei, Taiwan), En Chu Kong Hospital (ECKH; Sansia, Taiwan), Far Eastern Memorial Hospital (FEMH; Banciao, Taiwan), Chang Gung Memorial Hospital (CGMH; Linkou, Taiwan), Department of Medical Microbiology, University of Manitoba (Winnipeg, MB, Canada), and Department of Clinical Microbiology, Kuopio University Hospital (Finland) [19]. Together, strains representing the 78 known capsular types were included for *wzc* sequencing.

Between 2004 and 2006, Twenty-nine strains were collected from the blood of patients admitted to NTUH with bacteremia. To evaluate the *wzc* typing system in typing strains with unknown capsular types, all of the 29 *K. pneumoniae* clinical isolates of unknown capsular type were screened by *wzc* sequencing.

A *K. pneumoniae* clinical isolate from NTUH, NTUH-K2044 (K1), and its isogenic mutants NTUH-K2044 Δ*magA* (capsule deficient) and NTUH-K2044 Δ*wbbO* (O-antigen deficient) [32] were used as controls for Alcian blue staining.

### wzc sequencing

**Figure 1:**
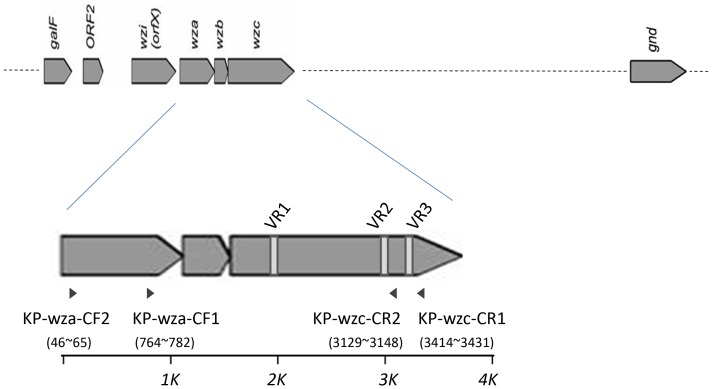
Variable regions VR1, VR2 and VR3, and the primers for PCR amplification of the *wza*-*wzb*-*wzc* region. The conserved genes of capsular polysaccharide synthesis (*cps*) region are shown as arrows. Variable regions (designated VR1, VR2, and VR3) of *wzc* are indicated as vertical white bars in the *wzc* ORF. Arrowheads indicate the positions and orientations of primers used for PCR amplification of the *wza*-*wzb*-*wzc* region. The positions of the primers in NTUH-K2044 were shown in brackets.

**Figure 2:**
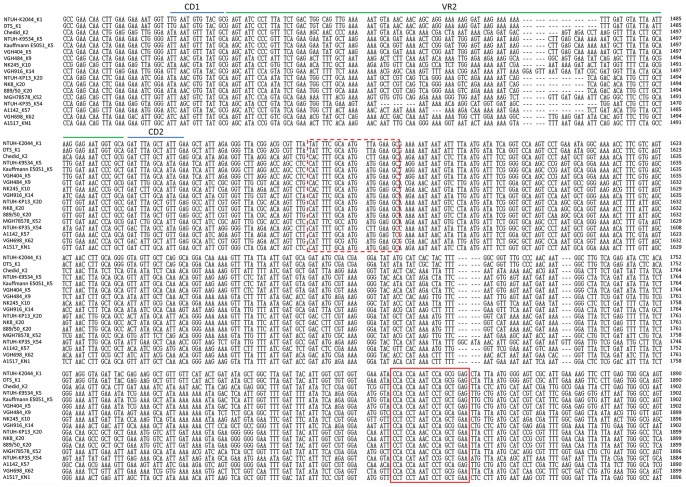
*wzc* sequence alignment across 12 capsular types. *wzc* sequences of 17 strains (NTUH-K2044, DTS, Chedid, NTUH-K9534, Kauffmann E5051, VGH404, VGH484, NK245, VGH916, NTUH-KP13, NK8, 889/50, MGH 78578, NTUH-KP35, A1142, VGH698 and A1517) representing 12 capsular types (K1, K2, K5, K9, K10, K14, K20, K52, K54, K57, K62, and KN1) were aligned with amino acid sequences and then back-translated to DNA sequences using MEGA 4.0 (ClustalW) with default parameters. The alignments of nucleotides number ∼1300–1900 of *wzc* ORF are shown in this figure. As shown by the alignment, two variable regions of *wzc* (∼1420–1480 and ∼1700–1820) are flanked by conserved regions. *wzc* sequences used in the design of reverse primers are marked by boxed domains (KP-wzc-CR1: continuous line box; KP-wzc-CR2: dashed line box).

Consensus sequences were identified based on the published *cps* sequences of 12 capsular types (K1, K2, K5, K9, K10, K14, K20, K52, K54, K57, K62, and KN1; sequences obtained from Genbank as Accession Numbers AB198423, AY762939, D21242, AB289646, AB371292, AB289645, AB371293, AB371291, AB371294, AF118250, AB289648, AB371289, CP000647, AB289650, AB334776, AB371295, and AB334777). The reference sequences were used to design forward primers KP-wza-CF1 and KP-wza-CF2 (corresponding to *wza* gene sequences) and reverse primers KP-wzc-CR1 and KP-wzc-CR2 (corresponding to *wzc* gene sequences), combinations of which were expected to permit PCR amplification of the *wza-wzb-wzc* region (Table 1, Figure 1, and Figure 2). Positions of these primers were shown in Figure 1 according to the sequences of NTUH-K2044. PCR amplifications were performed with the Long and Accurate PCR system (Takara, Tokyo, Japan). The cycling program was 96°C for 3 min, followed by 30 temperature cycles of 96°C for 30 s, 46°C for 15 s, and 72°C for 3 min. The expected size of PCR amplicons was ∼2.7 kb by use of primer pair 1 (KP-wza-CF1 and KP-wzc-CR1); ∼3.4 kb by use of primer pair 2 (KP-wza-CF2 and KP-wzc-CR1); ∼2.4 kb by use of primer pair 3 (KP-wza-CF1 and KP-wzc-CR2); ∼3.1 kb by use of primer pair 4 (KP-wza-CF2 and KP-wzc-CR2). PCR products were isolated and subjected to sequencing using reverse primers. In order to establish *wzc* database of documented capsular types, the amplicons were sequenced by primer walking using internal primers (Table S1) (each walk read ≧600 bp and there were at least 50 bp overlaps with previous obtained sequences). The obtained sequences were aligned from 96 strains representing 76 of the documented capsular types (Accession Numbers AB719985-AB720026, AB720650-AB720698, and AB819898). Amplification products were not obtained from strains representing types K15 and K50. The database is composed of *wzc* sequences (∼1.5 kb) from start codon of the *wzc* gene to the conserved domain CD2 (GANNTNNCNNTNNA) located in the downstream of VR2 region.

**Table 1:** Primers used in this study.

Primer name	Sequence	Purpose or reference
1166F	GGTGCTCTTTACATCATTGC	K1 genotyping [25]
936R	GCAATGGCCATTTGCGTTAG	K1 genotyping [25]
K2-wzyF	ATGATTCGAAGAAAGTTTTC	K2 genotyping
K2-wzyR	TTAGTTGATGTCATTTTCGG	K2 genotyping
K9-wzyF	ATGGTGATTATGAATGAAG	K9 genotyping
K9-wzyR	ACACAATGAAAACATTGCC	K9 genotyping
K14-wzyF	GACTCTGAATAAAAGAACAC	K14 genotyping
K14-wzyR	CTCAATAAATCTGTTCTGAAG	K14 genotyping
K15-wzyF	TACCCATAGCTATATGCGCC	K15 genotyping
K15-wzyR	GGGAAAGTTGCAGCATATTC	K15 genotyping
K16-wzyF	ATGGTACCGTTGGGGTTATC	K16 genotyping
K16-wzyR	TAATCAACAATGTCGTAGCG	K16 genotyping
K20-wzyF	GTGAGGACACTTTCGAAAGC	K20 genotyping
K20-wzyR	TCATTTACATTCCTTCTTCC	K20 genotyping
K23-wzyF	GTCATCTACTCTGTCCTTTTGGTCC	K23 genotyping
K23-wzyR	ATTACTATGTTGCGCCGAGG	K23 genotyping
K39-wzyF	ATGACCAATGACTTACAAAG	K39 genotyping
K39-wzyR	GAATTCCGTTCCAGCCCAC	K39 genotyping
K45-wzyF	GAACGAAGATGAACTCTGAC	K45 genotyping
K45-wzyR	GCTAGATTCGCATATGGAGA	K45 genotyping
K50-gly1F	CCAATGATAA TACTGCGCAG	K50 genotyping
K50-gly1R	CAACCCGATC ATATCATCTC	K50 genotyping
K54-F	TTACCTCAGAGCGTTGCATTG	K54 genotyping
K54-R	TTAGGTATGACAATTGAGCTC	K54 genotyping
K62-wzyF	ATGTCAGTGATTATTTCAGG	K62 genotyping
K62-wzyR	AGAGTATGTCATCACGCACG	K62 genotyping
N1-wzyF	TATGGGCTTAGGTTTCCTGG	KN1 genotyping
N1-wzyR	TGCAATATAAATCTCCCCAG	KN1 genotyping
1461-wzyF	GCAGAATTCGATAGCTTGTC	1461 genotyping
1461-wzyR	CCGCATAAACCATGTCATTG	1461 genotyping
KP-wza-CF1	TGAAAGTGTTTGTCATGGG	*wzc* PCR
KP-wza-CF2	GGGTTTTTATCGGGTTGTAC	*wzc* PCR
KP-wzc-CR1	TTCAGCTGGATTTGGTGG	*wzc* PCR
KP-wzc-CR2	GCTTCCATCATTGCAAAATG	*wzc* PCR
K22-acyl-F	TATCCATATGCTGTTTGGTC	Acetyltransferase sequencing
K22-acyl-R	TCGCAGCGGTATACAAATTC	Acetyltransferase sequencing

Note: primers used for sequencing were shown in a supplementary table (Table S1).

### Sequencing of cps region

Since we failed to amplify *wzc* genes from reference strains K15 and K50, we instead amplified the *cps* region from these strains using conserved primers CPS-1 (located in the *wzi* gene) and rCPS (located in *gnd*), as previously described [19]. To permit comparison among the *cps* regions of selected strains, the corresponding regions were amplified from strains K22, K37, K45, K79, and novel type strain 1461 using primers CPS-1 and rCPS as well. PCR amplifications were performed with the Long and Accurate PCR system. The cycling program consisted of one denaturation step of 2 min at 94°C and 10 initial cycles of 10 s at 98°C, 30 s at 63°C, and 12 min at 68°C, followed by 20 iterative cycles of 10 s at 98°C, 30 s at 63°C, and 12 min plus 20 s for each new cycle at 72°C. A final elongation step was performed for 10 min at 72°C. To extend upstream and downstream from the conserved regions (from *galF* to *gnd*), primers pre-galF-F and yegH (located in the sequences at the upstream end of *cps*) and post-gnd R and ugd (located in the sequences at the downstream end of *cps*) were used to amplify the flanking sequences [19]. The PCR cycling program for these reactions consisted of 96°C for 3 min, followed by 30 cycles of 96°C for 30 s, 52°C for 15 s, and 72°C for 2–5 min. The products were sequenced by primer walking, providing complete sequences for the *cps* regions (from *galF* to *gnd*, extending approximately 20 kb). The resulting sequences were deposited to Genbank as Accession Numbers AB819892-AB819894, AB819896, AB819897, AB819895, and AB822494). Genes were annotated by NCBI-blast.

### wzy-PCR genotyping

To confirm the capsular types of clinical isolates, primers located in the capsular type-specific *wzy* gene in variable region of *cps* loci were used. Along with the published *wzy* genes of types K1, K2, K14, K20, K54, K62, and KN1 (Accession Numbers AB198423, D21242, AB371294, AB289648, AB289650, AB371295, AB334777), we also resolved sequences for the *cps* regions of reference strains for types K16, K23, and K39 (Accession Numbers AB742228, AB742229, and AB742230). Specifically, we designed specific *wzy* primers based on the sequences for *cps*-PCR genotyping (Table 1). PCR was performed as previously described [19].

Primers specific for the *wzy* gene of strain 1461 were designed (Table 1) with the intent of confirming the presence of *cps* genes distinct from the 78 documented capsular types. In parallel to PCR with strain 1461, *cps*-PCR genotyping using the same primers was performed in 77 K-serotype reference strains (Statens Serum Institute) and KN1 (A1517). Primers pair 1461-wzyF and 1461-wzyR were used in 1461 *wzy-*PCRgenotyping.

### Alcian blue staining

Extracellular polysaccharides, including both capsule and lipopolysaccharide, were isolated as previously reported [33]. Briefly, bacteria were cultured overnight in 1 mL Luria-Bertani (LB) medium and then harvested and resuspended in 150 µL of water. An equal volume of phenol (pH 6.6; Amresco) was added, and the mixture was vortexed. After incubation at 65°C for 20 min, samples were extracted with chloroform and centrifuged. The extracted samples were separated by 10%-sodium dodecyl sulfate-polyacrylamide gel electrophoresis (SDS-PAGE) and capsule was detected with Alcian blue as previously described [34], [35]. In brief, after electrophoresis, the gel was washed three times (5 min, 10 min, and 15 min; at 50°C for each step) with fix/wash solution (25% ethanol, 10% acetic acid in water). The gel then was soaked (15 min in the dark at 50°C) in 0.125% Alcian blue dissolved in fix/wash solution, and finally destained (overnight at room temperature) with fix/wash solution. CPS was visualized as blue-stained material.

## Results

### wzc sequences are distinct in different capsular types

**Figure 3:**
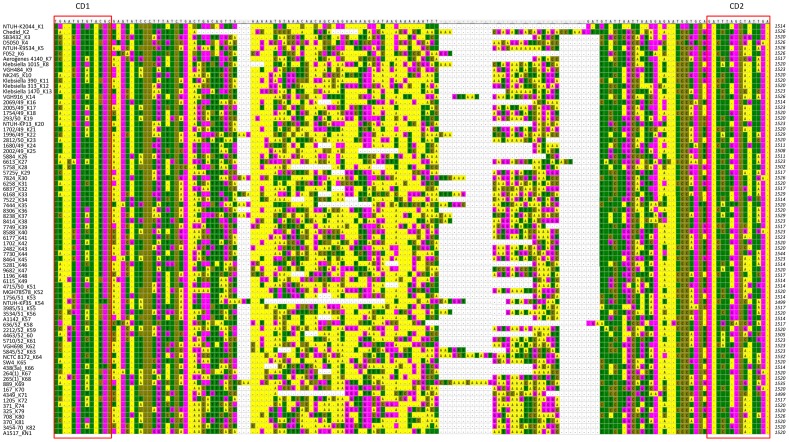
Variable region 2 (VR2) of *wzc* and conserved domain (CD1 and CD2) in 76 capsular types. VR2 and the nearby sequences of 76 capsular types were shown. Nucleotides above the alignment indicate K1 sequences and conserved bases identical to K1 were shown as dots. Cells were in different colors according to the nucleotides (A: yellow; T: green; C: brown; G: pink). CD1 (TNANNGTNTANNC) and CD2 (GANNTNNCNNTNNA) marked by opened square were conserved among all 76 capsular types.

The capsular polysaccharide synthesis (*cps*) region of *K. pneumoniae* shows conserved genetic organization (synteny) extending from *galF* through *orf2*, *wzi*, *wza*,*wzb*, *wzc*, and *gnd*. Genes between *wzc* and *gnd* (downstream of *wzc* to upstream of *gnd*) vary among different capsular types and therefore are considered to constitute a variable region (Figure 1). We compared the conserved genes (*galF*, *orf2*, *wzi*, *wza*, *wzb*, *wzc*, and *gnd*) among the published *cps* sequences of 17 strains with 12 capsular types (K1, K2, K5, K9, K10, K14, K20, K52, K54, K57, K62, and KN1) and noted that the *wzc* genes (encoding a tyrosine kinase) had three variable regions flanked by more conserved regions. The sequences of variable regions were highly variable among different capsular types but were conserved in the strains with same capsular type. The *wzc* genes were aligned with amino acid sequences and then back-translated into codons. These variable regions of *wzc* (which we refer to as VR1, VR2, and VR3) mapped to nucleotides ∼420–480, ∼1420–1480, and ∼1700–1820 (respectively) of the ORF from different capsular types (The position of VR1, VR2 and VR3 in NTUH-K2044 was ∼423–460, ∼1422–1480 and ∼1713–1801 respectively). Moreover, the domains at ∼1480–1660 and∼1820–1860 bases showed DNA sequence conservation (The position of the conserved domain in NTUH-K2044 was ∼1489–1634, and ∼1828–1852 respectively). Based on these sequences, we designed consensus reverse primers KP-wzc-CR1 and KP-wzc-CR2 (Figure 1 and Figure 2) (The position of KP-wzc-CR1 and KP-wzc-CR2 in NTUH-K2044 were 1828–1845 and 1543–1562 of the *wzc* gene, respectively). Similar analysis of the *wza* genes in the 12 capsular types permitted the design of forward primers KP-wza-CF1 and KP-wza-CF2 (Figure 1) (The position of KP-wza-CF1 and KP-wza-CF2 in NTUH-K2044 were 764–782 and 46–65 of the *wza* gene, respectively). In order to complete the *wzc* sequences database of 78 documented capsular types, the primers (in each of the four primer pair combinations) were used to amplify the *wza-wzb-wzc* region (∼2.5–3 kb) from a total of 81 strains with known capsular types (Table 2). The PCR results revealed that primer pair 1 (KP-wza-CF1 and KP-wzc-CR1), primer pair 2 (KP-wza-CF2 and KP-wzc-CR1), primer pair 3 (KP-wza-CF1 and KP-wzc-CR2), and primer pair 4 (KP-wza-CF2 and KP-wzc-CR2) amplified fragments from 92%, 88%, 82%, and 81% of the documented capsular types, respectively. The combination of primer pair 1 and primer pair 2 yielded products from 76 of the 78 documented capsular types (97%). The exceptions were capsular type K15 and K50 strains, which did not yield product by any of the four primer pairs. PCR products amplified by primer pair 1 were sequenced by the reverse primer KP-wzc-CR1 in 75 of the 81 strains excluding reference strains of K15, K32, K50, K59, K67 and K79; PCR products amplified by primer pair 2 were sequenced by the reverse primer KP-wzc-CR1 in reference strains K32, K59, K67 and K79. The PCR amplicons were sequenced to the start codon of *wzc* gene by primer walking. All of the sequences deposited in Genbank were from start codon of *wzc* to CD2 domain (GANNTNNCNNTNNA) which is located in the downstream of VR2 (Figure 3) and exhibit conservation among 76 capsular types. Thus, the sequences obtained from published *cps* sequences and from our sequencing results together constitute a 96-strain database of *wzc* sequences (Table 2). Comparison among the amino acid and DNA sequences of *wzc* revealed high levels of similarity (≧99% identity both by amino acid sequences and DNA sequences) derived from strains with same capsular type. Strains belonging to distinct capsular types exhibited lower levels of similarity (40–80% identity by amino acid sequences and 60–80% identity by DNA sequences), with three exceptions. Specifically, the K22 and K37 type strains had *wzc* sequences that were identical to each other; the K9 and K45 type strains shared 99% amino acid or DNA sequence identity; and the K52 and K79 type strains shared 93% amino acid sequence identity (89% DNA sequence identity). In order to make the method more easily to be used for capsular type identification, conserved regions, CD1 (TNANNGTNTANNC) and CD2 (GANNTNNCNNTNNA), nearby VR2 were identified in 76 capsular types. The CD1-VR2-CD2 region (115–151 bp in length from different capsular types) was selected for comparison (Figure 3 and File S1). Therefore, only one-run sequencing using KP-wzc-CR1 (∼350 bp from CD2) or KP-wzc-CR2 (∼60 bp from CD2) can cover this region for further comparison. The CD1-VR2-CD2 region from distinct capsular types in our *wzc* database showed <80% DNA identity and the region derived from strains with same capsular type shared ≧97% DNA identity with the exception of K22/K37 (142/142, 100%), K9/K45 (133/136, 98%) and K52/K79 (123/136, 90%).

**Table 2:** Strains included in the *wzc* database established in this study.

Capsular type	Strain
K1	NTUH-K2044^a^ (AB719985), DTS^a^ (AY762939)
K2	Chedid^a^ (AB719986)
K3	SB3432^a^ (AB719987)
K4	D5050^b, c^ (AB719988)
K5	NTUH-K9534^a^ (AB719989), Kauffmann E5051^a^ (AB289645), VGH404^a^ (AB371292), E6^b^ (AB719990), can0525^b^ (AB719991)
K6	F052^b, c^ (AB719992)
K7	Aerogenes 4140^b, c^ (AB719993)
K8	Klebsiella 1015^b, c^ (AB719994)
K9	VGH484^ a^ (AB719995), ATCC29013^b^ (AB719996)
K10	NK245^a^ (AB719997), Klebsiella 919^b, c^ (AB719998)
K11	Klebsiella 390^b, c^ (AB719999)
K12	Klebsiella 313^b, c^ (AB720000)
K13	Klebsiella 1470^b, c^ (AB720001)
K14	VGH916^a^ (AB720002), Klebsiella 1193^b, c^ (AB720003)
K15	N/A
K16	2069/49^b, c^ (AB720004), can0416^b^ (AB720005), N4795^b^ (AB720006)
K17	2005/49^b, c^ (AB720007)
K18	1754/49^b, c^ (AB720008)
K19	293/50^b, c^ (AB720009)
K20	NTUH-KP13^a^ (AB720010), NK8^a^ (AB720011), 889/50^ a^ (AF118250)
K21	1702/49^b, c^ (AB720012)
K22	1996/49^b, c^ (AB720013)
K23	2812/50^b, c^ (AB720014)
K24	1680/49^b, c^ (AB720015)
K25	2002/49^b, c^ (AB720016)
K26	5884^b, c^ (AB720017)
K27	6613^b, c^ (AB720018)
K28	5758^b, c^ (AB720019)
K29	5725y^b, c^ (AB720020)
K30	7824^b, c^ (AB720021)
K31	6258^b, c^ (AB720022)
K32	6837^b, c^ (AB720023)
K33	6168^b, c^ (AB720024)
K34	7522^b, c^ (AB720025)
K35	7444^b, c^ (AB720026)
K36	8306^b, c^ (AB720650)
K37	8238^b, c^ (AB720651)
K38	8414^b, c^ (AB720652)
K39	7749^b, c^ (AB720653)
K40	8588^b, c^ (AB720654)
K41	6177^b, c^ (AB720655)
K42	1702^b, c^ (AB720656)
K43	2482^b, c^ (AB720657)
K44	7730^b, c^ (AB720658)
K45	8464^b, c^ (AB720659)

Capsular type	Strain
K46	5281^b, c^ (AB720660)
K47	9682^b, c^ (AB720661)
K48	1196^b, c^ (AB720662)
K49	6115^b, c^ (AB720663)
K50	N/A
K51	4715/50^b, c^ (AB720664)
K52	MGH78578^a^ (AB720665), 5759/50^b, c^ (AB819898)
K53	1756/51^b, c^ (AB720666)
K54	NTUH-KP35^a^ (AB720667), can0405^b^ (AB720668), 4538E^b^ (AB720669), B33^b^ (AB720670), E8^b^ (AB720671)
K55	3985/51^b, c^ (AB720672)
K56	3534/51^b, c^ (AB720673)
K57	A1142^a^ (AB720674), 4425/51^b, c^ (AB720675), H14^b^ (AB720676), Finland^b^ (AB720677)
K58	636/52^b, c^ (AB720678)
K59	2212/52^b, c^ (AB720679)
K60	4463/52^b, c^ (AB720680)
K61	5710/52^b, c^ (AB720681)
K62	VGH698^a^ (AB720682)
K63	5845/52^b, c^ (AB720683)
K64	NCTC 8172^b, c^ (AB720684)
K65	SW4^b, c^ (AB720685)
K66	438(3a)^b, c^ (AB720686)
K67	264(1)^b, c^ (AB720687)
K68	265(1)^b, c^ (AB720688)
K69	889^b, c^ (AB720689)
K70	167^b, c^ (AB720690)
K71	4349^b, c^ (AB720691)
K72	1205^b, c^ (AB720692)
K74	371^b, c^ (AB720693)
K79	325^b, c^ (AB720694)
K80	708^b, c^ (AB720695)
K81	370^b, c^ (AB720696)
K82	3454-70^b, c^ (AB720697)
KN1	A1517^a^ (AB720698)

a, sequences were obtained from Genbank; b, sequences were resolved in this study; c, reference strain. N/A, not available. Accession numbers were shown in brackets.

### K15 and K50 were found to have transposase insertions that precludes capsule expression

**Figure 4:**
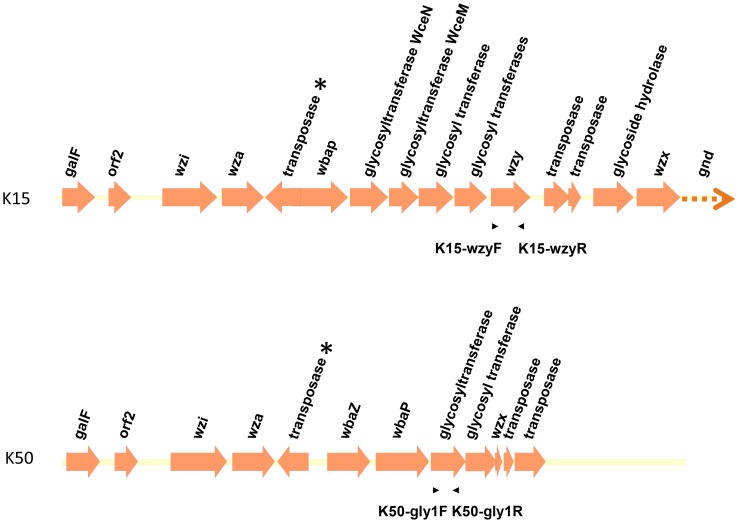
Genetic alignment of K15 and K50 *cps* regions. Open reading frames (ORFs) are shown as arrows. The arrows with dotted lines indicate that only partial sequences were obtained for these ORFs. Asterisks indicate the genes encoding putative transposases that replace the *wzb* and *wzc* genes in the K15 and K50 strains. The positions and orientations of primers used for *cps*-PCR genotyping are indicated by arrow heads. Primer pair K15-wzyF and K15-wzyR was used for K15 *cps*-PCR genotyping, and primer pair K50-gly1F and K50-gly1R was used for K50 *cps*-PCR genotyping.

As noted above, PCR amplification of the ∼2.5–3 kb *wza-wzb-wzc* region using the *wza* and *wzc* primers failed in reference strains K15 and K50. We therefore amplified and sequenced the full *cps* region by PCR. The resulting sequences (Accession Numbers AB819895 and AB822494) revealed that both the *wzb* and *wzc* genes were replaced by genes encoding transposases both in K15 and K50 (Figure 4). We further designed additional specific primer pairs based on the sequences of the *wzy* gene of K15 (primers K15-wzyF and K15-wzyR) and the sequences of a gene encoding a glycosyltransferase homolog in K50 (primers K50-gly1F and K50-gly1R) (Table 1 and Figure 4). PCR performed on each of the 78 capsular type strains confirmed that these primers were specific for the K15 and K50 capsular types (data not shown). Therefore, although *wzc* genotyping was not successful for capsular type K15 and K50, type-specific primers can be used to genotype the K15 and K50 strains.

**Figure 5:**
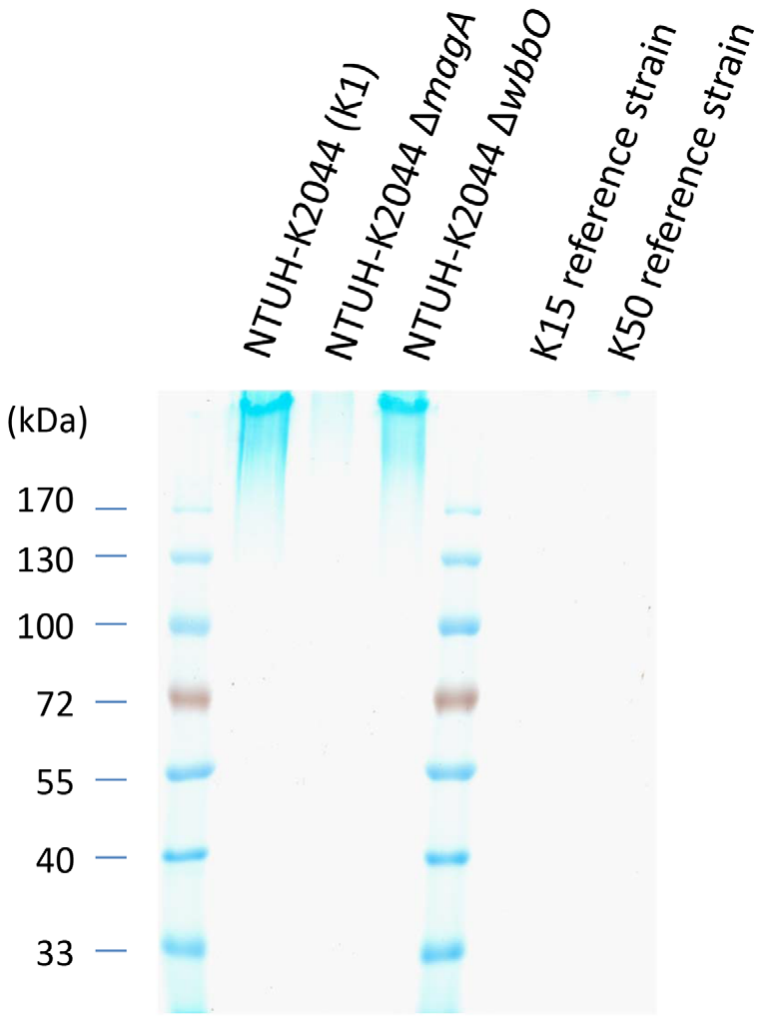
Alcian blue staining of polysaccharide extracts from reference strains K15 and K50. Polysaccharide extracts from NTUH-K2044 (K1), isogenic mutant NTUH-K2044 Δ*magA* (capsule deficient), isogenic mutant NTUH-K2044 Δ*wbbO* (O-antigen deficient), and reference strains K15 and K50 were stained with Alcian blue (see Materials and Methods).

Moreover, the *wzb* and *wzc* genes are thought to be essential for capsule synthesis in *Klebsiella*, suggesting the loss of capsule in the K15 and K50 strains. Therefore, we used Alcian blue staining to determine the capsular status of these strains. Our results revealed the absence of CPS in reference strains K15 and K50, as also seen with NTUH-K2044 Δ*magA*, a known capsule-deficient mutant; in contrast, CPS (visualized as high-molecular weight Alcian blue stained material at the top of an SDS-PAGE gel) was observed in positive controls, including a K1 strain (NTUH-K2044) and an isogenic Δ*wbbO* (O-antigen-deficient) mutant (Figure 5). Thus, the reference strains K15 and K50 are acapsular.

### cps regions of K22/K37, K9/K45, and K52/K79

**Figure 6:**
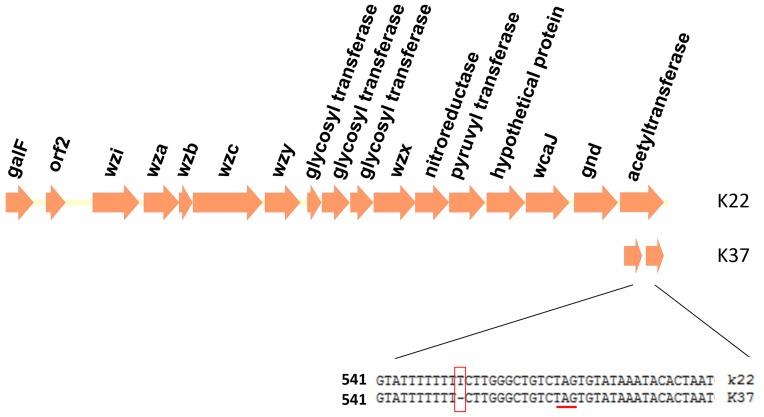
Genetic alignment of K22 and K37 *cps* regions and a frameshift mutation in K37. Open reading frames (ORFs) are shown as arrows. In K37, a single nucleotide deletion (boxed) was observed at nucleotide position 551 of a gene encoding a putative acetyltransferase. This deletion is predicted to result in truncation (at the underlined stop codon) of the ORF in K37.

As noted above, sequencing of *wzc* revealed higher than expected DNA sequence similarities between the type strains for K22 and K37 (100% identity at *wzc*), K9 and K45 (99% identity), and K52 and K79 (89% identity). We therefore further explored the genetic structure of the *cps* regions in these strains. The sequences (Accession Numbers AB819893 and AB819894) showed that K22 and K37 not only have the same *wzy* gene which is thought to be distinct among different capsular types, but also have indistinguishable *cps* regions with the exception of a sequence difference in the ORF downstream of *gnd*. In K22, this ORF encodes a putative acetyltransferase; in K37, the ORF is truncated as a result of a frameshift mutation (single nucleotide deletion) relative to K22 (Figure 6). Interestingly, this result is consistent with the previous finding that the capsule structures of K22 and K37 differ only by the presence of acetyl group in K22 CPS [36]. We designed two primers (K22-acylF and K22-acylR) appropriate for amplification of the acetyltransferase gene (Table 1). Sequencing of the resulting amplicon is expected to reveal the status of the putative acetyltransferase-encoding gene, permitting the distinction between K22 and K37 despite identity in both *wzc* and *wzy*.

**Figure 7:**
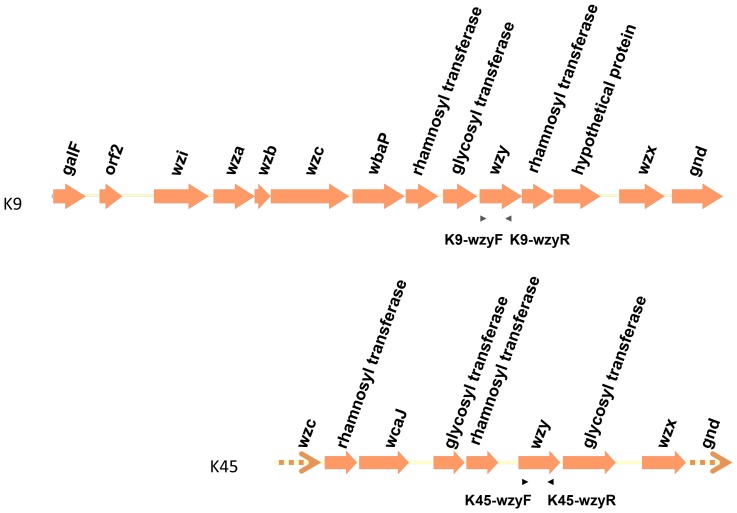
The genetic alignment of K9 and K45 *cps* regions and the primers for *cps*-PCR genotyping. Open reading frames (ORFs) are shown as arrows. The arrows with dotted lines indicate that only partial sequences were obtained for these ORFs. The positions and orientations of *cps*-PCR genotyping primers, located in the *wzy* genes, are indicated by arrow heads. Primer pair K9-wzyF and K9-wzyR was used for K9 *cps*-PCR genotyping; primer pair K45-wzyF and K45-wzyR was used for K45 *cps*-PCR genotyping.

Although the *wzc* genes of K9 and K45 showed high DNA sequences similarity (99% identity), genes located in the *cps* regions differed between these two capsular types (Figure 7; Accession Numbers AB371293 and AB819892). We designed primers K9-wzyF, K9-wzyR, K45-wzyF, and K45-wzyR based on the sequences of the *wzy* genes of K9 and K45 (Table 1 and Figure 7), and demonstrated that PCR amplification with K9-wzyF and K9-wzyR was detected in the K9 capsular type strain but not in the other 77 capsular type strains. Likewise, K45-wzyF and K45-wzyR also showed specificity for capsular type K45 (data not shown). Therefore, these two type-specific primer pairs can be used to distinguish K9 and K45, despite highly similar *wzc* sequences.

**Figure 8:**
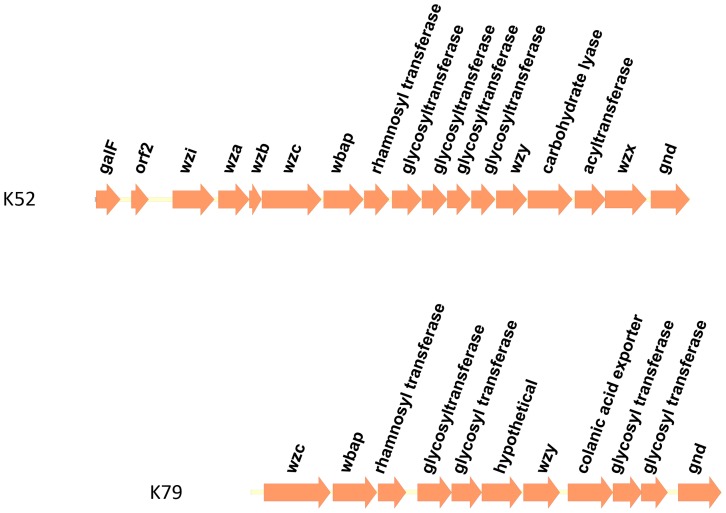
The genetic alignment of the K52 and K79 *cps* regions. Open reading frames (ORFs) are shown as arrows.

The full sequenced *cps* regions of K52 and K79 type strains revealed that these type strains possessed different genes in the clusters, although the strains shared 89% identity in *wzc* sequences (Figure 8; Accession Numbers CP000647 and AB819896). Furthermore, the *wzc* sequences of the two K52 strains in our panel exhibited 100% identity at the DNA level, suggesting that K52 and K79 can still be distinguished despite similarities in *wzc* sequences.

### wzc genotyping of clinical isolates with unknown capsular types

**Figure 9:**
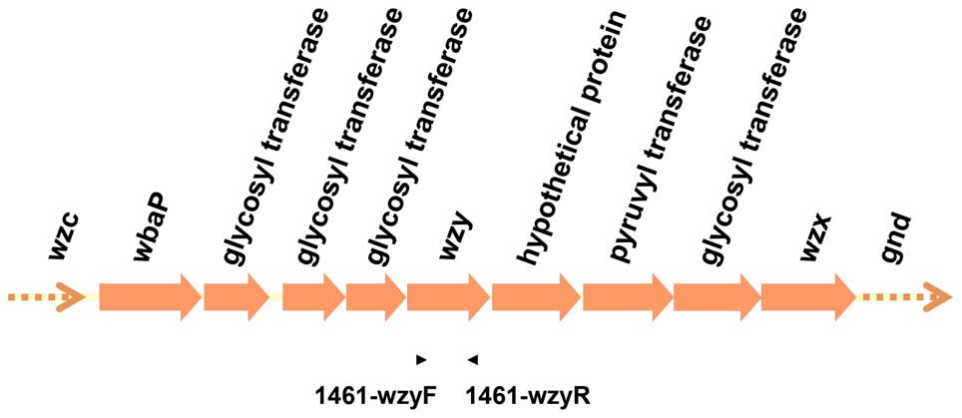
The genetic alignment of *cps* region of a probable new type and primers for *cps*-PCR genotyping. Open reading frames (ORFs) are shown as arrows. The arrows with dotted lines indicate that only partial sequences were obtained for these ORFs. The positions and orientations of *cps*-PCR genotyping primers, located in the *wzy* gene, are indicated by arrow heads. Primer pair 1461-wzyF and 1461-wzyR was used for 1461 *cps*-PCR genotyping.

To evaluate the *wzc* genotyping system, capsular types of 29 *K. pneumoniae* blood isolates (obtained from patients admitted to NTUH) were determined by our method. The four primer pairs described above were used for PCR amplifications of the *wza*-*wzb*-*wzc* regions of these strains. The four primer pairs provided typing by PCR amplification in 90% (26/29) of these strains using primer pair 1 (KP-wza-CF1 and KP-wzc-CR1), 97% (28/29) using primer pair 2 (KP-wza-CF2 and KP-wzc-CR1), 59% (17/29) using primer pair 3 (KP-wza-CF1 and KP-wzc-CR2), and 62% (18/29) using primer pair 4 (KP-wza-CF2 and KP-wzc-CR2). The combination of primer pairs 1 and 2 permitted typing of all 29 of the tested strains. The amplified PCR products by use of primer pairs 1 were sequenced by the reverse primer KP-wzc-CR1 in 26 of the 29 strains, whereas the primer pair 2-amplicons were subjected to sequencing with KP-wzc-CR1 in the remaining three strains, my1684, 5872, and 5982-2. Sequences from CD1 to CD2 covered VR2 region (115-151 bp) were used for comparing with our *wzc* database. The results revealed that among the 29 strains, 28 strains showed high DNA sequence similarity (≧94% identity) with documented capsular types in CD1-VR2-CD2 region. Based on the DNA sequences, these 28 strains were classified as capsular type K1 (n = 6), K2 (5), K16 (3), K20 (2), K54 (2), K28 (2), K14 (1), K23 (1), K24 (1), K27 (1), K39 (1), K61 (1), K62 (1), and KN1 (1). We further confirmed the results by *wzy*-PCR genotyping using type-specific *wzy* primers for K1, K2, K14, K16, K20, K23, K39, K54, K62, and KN1 [19], [25]. The results demonstrated that *wzc* genotyping provided results consistent with *wzy* genotyping (Table 3). The one (out of 29) remaining strain showed relatively low DNA sequence similarity in CD1-VR2-CD2 region (<70% identity) with the documented capsular types in our *wzc* panel, suggesting that this strain represented a novel *wzc* sequence. Therefore, we further evaluated this strain to determine whether this strain represented a new capsular type distinct from the previously described 78 types. Specifically, the *cps* region of strain 1461 was amplified and the variable regions of *cps* gene cluster was analyzed (Figure 9) (Accession Number AB819897). Specific primers 1461-wzyF and 1461-wzyR were designed based on the novel *wzy* sequence (Table 1 and Figure 9); PCR genotyping with this primer pair provided detection only for strain 1461, and not for any of the 78 documented capsular types (data not shown). Based on these results, we infer that this strain likely represents a novel capsular type.

**Table 3:** *wzc* type of *K. pneumoniae* clinical isolates causing bacteremia.

Strain	*wzc* type	DNA sequences identity (%)	*wzy* PCR check
526	K1	124/124(100%), NTUH-K2044	K1 +
1024	K1	124/124(100%), NTUH-K2044	K1 +
229	K1	124/124(100%), NTUH-K2044	K1 +
217	K1	124/124(100%), NTUH-K2044	K1 +
9285	K1	124/124(100%), NTUH-K2044	K1 +
92	K1	124/124(100%), NTUH-K2044	K1 +
7313	K2	133/133(100%), Chedid	K2 +
1529	K2	133/133(100%), Chedid	K2 +
1730	K2	133/133(100%), Chedid	K2 +
5154	K2	133/133(100%), Chedid	K2 +
9951-2	K2	133/133(100%), Chedid	K2 +
4410	K14	142/142(100%), K14 ref	K14 +
7476	K16	124/124(100%), K16 ref	K16+
7270	K16	124/124(100%), K16 ref	K16+
4001-2	K16	122/124(98%), K16 ref	K16 +
5262	K20	127/127(100%), NTUH-KP13	K20 +
1296	K20	120/127(94%), NTUH-KP13	K20 +
3329	K23	133/133(100%), K23 ref	K23+
8531	K24	124/124(100%), K24 ref	N/A
8577	K27	139/139(100%), K27 ref	N/A
my1684	K28	115/115(100%), K28 ref	N/A
5982-2	K28	115/115(100%), K28 ref	N/A
6737	K39	133/133(100%), K39 ref	K39 +
3200	K54	115/115(100%), NTUH-KP35	K54+
5872	K54	114/115(99%), NTUH-KP35	K54 +
6257	K61	136/136(100%), K61 ref	N/A
6341	K62	136/136(100%), VGH698	K62 +
8393	KN1	133/136(98%), A1517	KN1 +
1461	new	(wzc database <70%)	N/A

Note: the ratio of identity indicates no. of matching nucleotides/total no. of nucleotides of the CD1-VR2-CD2 region from the strain with highest similarity in our *wzc* database.

## Discussion

Serotyping has been used for determination of *K. pneumoniae* K-types since 1926 [37]. However, several studies have suggested that a substantial proportion (ranging from 23% to 75% in different laboratories) of strains are non-typable by serotyping. [20], [22], [23]. These observations could reflect limited assay sensitivity, or could reflect limited assay specificity (e.g., serological cross-reactivity between different capsular types). In addition, the high cost and limited sources of anti-sera and tedious experimental procedures of serotyping make the practice of serotyping difficult. Therefore, capsular genotyping methods that bypass the use of anti-sera have become more widely used in discriminating the capsular types of *K. pneumoniae* [6], [7], [19], [25]–[30]. PCR-based *cps* genotyping is a rapid and accurate method for detecting *cps* genotype [25]. Since the gene layout and DNA sequences of variable regions in the *cps* synthesis loci are distinct in different capsular types, type-specific primers (located in *wzy*-like genes or other genes of the *cps* gene cluster) can be used for distinguishing capsular types. However, this method does not permit detection of all capsular types, because classification cannot be performed unless the DNA sequences of the entire *cps* gene cluster are available. One study reported a novel capsular genotyping method, *cps* PCR-RFLP analysis, that permitted typing with high discriminatory power [31]. In this method, capsular types are determined according to the distinct RFLP profiles (C-patterns). In addition, this technique permits distinction among strains with the same K serotype, because subtle differences in DNA sequences can be detected based on variations in *cps* PCR-RFLP pattern. However, this increased complexity may complicate interpretation of capsular genotyping. Moreover, these two capsular genotyping methods (*cps-*PCR genotyping and *cps* PCR-RFLP) require the amplification of the entire ∼20 kb capsule synthesis region; such long PCR products can be difficult to obtain. By comparison, the *wzc* genotyping method (developed in the present study) requires amplification of a ∼2.5–3 kb PCR fragment and ∼350 bp of DNA sequencing can cover the variable region for comparison. As demonstrated by our PCR analysis of 78 capsular type strains, along with multiple clinical isolates, PCR amplicons were obtained in more than 90% of strains screened with primer pair 1 alone, and in up to 100% of strains screened with the combination of primer pairs 1 and 2. Therefore, our method is expected to be convenient and useful in clinical settings; most isolates will be identifiable using only one or two primer pairs, with few strains requiring testing with additional primers.

Our results indicated that *wzc* CD1-VR2-CD2 sequences were highly similar (≧94% DNA identity) among strains with the same capsular type. Relatively low levels of similarity (<80% identity) were observed among strains of different capsular types, with the exceptions of K22/K37 (100% *wzc* identity), K9/K45 (98% identity), and K52/K79 (90% identity). Since K52 and K79 can still be discriminated based on differences in *wzc* sequences, our proposed typing method is expected to discriminate 74 types (including type K22/K37 and K9/K45). Therefore, only the differentiation between types K22 and K37 (requiring sequencing of the putative acetyltransferase-encoding gene), between K9/K45 (requiring *cps*-PCR genotyping) and in *wzc*-deficient K15 and K50 (requiring *cps*-PCR genotyping) would require the use of additional assays.

After the *cps* regions of K22 and K37 were resolved, we found that K22 and K37 shared same *cps* genes for their capsule synthesis. This result was consistent with previous observation that the *cps* PCR-RFLP patterns of K22 and K37 were indistinguishable [38] and that K22 and K37 were usually cross-reactive by serotyping [39]. Interestingly, we also observed the truncation of a putative acetyltransferase-encoding ORF in K37 compared to K22, providing an explanation for the virtually identical (except for an acetylation modification) capsule structures of K22 and K37 [36]. This phenomenon is similar to that of pneumococcus 9V and 9A, which differ from each other only in the acetylation of capsule. Serotype 9V was found to possess an intact acetyltransferase-encoding gene, while the equivalent gene of serotype 9A was disrupted by a frameshift mutation (deletion of guanine at nucleotide 726) [40].

Although *wzc* sequences were almost identical in K9 and K45, the *cps* gene clusters differed between the two capsular types. This could be due to recombination in the region from *wzc* to *gnd*, resulting in gene replacement across this interval. Therefore, the *cps* sequence similarities between K9 and K45 and between K22 and K37 may provide insights into the evolution and divergence of capsular types.

The *cps* sequences of reference strains K15 and K50 revealed the presence in the clusters of several genes encoding transposase homologs. Notably, the typical *wzb*-*wzc* locus of the *cps* region was replaced by transposase-like genes in these two capsular types. The *wzb* and *wzc* genes may have been lost during chromosomal rearrangements associated with transposition events. Wzc, a tyrosine autokinase, is dephosphorylated by its cognate phosphatase, Wzb. Wza, located in the outer membrane, is known to interact with the periplasmic domain of Wzc and is believed to act as a channel [41]. These gene products (Wza, Wzb, and Wzc) are associated with the control of capsule polysaccharide polymerization and cross-membrane translocation, and are thought to be essential for capsule synthesis in *E. coli* and *Klebsiella* sp. [42]–[44]. We demonstrated that reference strains K15 and K50 were in fact acapsular. This observation is consistent with the absence of *wzb* and *wzc* in these two strains. Capsule structures of reference strains K15 and K50 from the same origin (Statens Serum Institute) have been reported in previous studies in 1992 and 1982 respectively [45], [46]. Our reference strains K15 and K50 were purchased from Statens Serum Institute in 2004 and stored at –80°C. Experiments in this study were performed using original stock in our laboratory, therefore, these two strains seemed to have lost capsule before we obtained them.

Using our proposed *wzc* typing method, we were able to successfully determine the capsular types of all of the clinical isolates tested, with the exception of a single strain that appears to represent a new capsular type. According to the comparison of sequences in our *wzc* database, strains with same capsular type shared ≧97% identity, but one strain among the clinical isolates of known types did not hit 97% identity. However, even though the only exception revealed 94% (<97%) DNA identity in CD1-VR2-CD2 region with the corresponding locus of capsular type K20 from our *wzc* database, *wzy* PCR genotyping confirmed that this strain was type K20. Therefore, our data suggest that strains harboring *wzc* CD1-VR2-CD2 sequences of ≧94% DNA sequence identity can be expected to share the same capsular type. Furthermore, the consistency of the results between *wzc*- and *wzy*-genotyping suggests that *wzc* should provide genotyping as accurate as that of *wzy*. Since not all of the *wzy* genes for the documented capsular types are currently available, *wzc* genotyping, a simple alternative method, may be more useful for complete capsular typing. Moreover, we infer that strains with novel *wzc* sequences probably represent new *cps* genotypes. Consistent with this hypothesis, we noted that the *cps* region of strain 1461 was distinct from those of previously reported capsular types. Notably, the *cps* gene cluster of strain 1461 was most similar to that of *E. coli* MS 146-1(Accession No. ADTN00000000). Previous studies had reported that *Klebsiella* K20 and *E. coli* K30 harbor identical capsule structures and highly similar *cps* sequences, implying that horizontal gene transfer had occurred between these strains [47]. Our results with strain 1461 provided further evidence for this phenomenon.

Wzc, an inner membrane protein with a cytosolic C-terminal tyrosine autokinase domain, is believed to interact with the outer membrane protein Wza, forming a trans-envelope capsule translocation complex. In the current study, we demonstrated capsular type-specific regions in the *wzc* locus. And we also found that VR2 region is rich in lysine (a basic amino acid). Therefore, the variable regions of *wzc* genes might encode binding domains containing positively charged amino acids. The lysine-rich domains might interact with type-specific acidic capsular polysaccharides during the process of translocation.

In conclusion, we have developed a simple and useful capsular genotyping method for *K. pneumoniae* based on *wzc* sequences. We demonstrated the use of this typing method for the detection of existing and novel capsular types of *K. pneumoniae*. Sequencing of *cps* loci suggested a molecular basis (frameshift mutation) for the difference between types K22 and K37, and revealed that reference strains K15 and K50 were acapsular.

## Supporting Information

Table S1 **Primers used for** ***wzc*** **sequencing.**
(DOCX)

File S1 **CD1-VR2-CD2 sequences.**
(TXT)
