# Supplementary material for: Capsular Types of Klebsiella pneumoniae Revisited by wzc Sequencing
Source: PLoS One. 2013 Dec 9;8(12):e80670. doi: 10.1371/journal.pone.0080670 (PMC3857182; doi:10.1371/journal.pone.0080670)
Supplement: Table S1 — Primers used for wzc sequencing. (DOCX) [file pone.0080670.s001.docx]

Table S1. Primers used for wzc sequencing

| Primer | Sequences | Purpose |
| --- | --- | --- |
| K4 R1 | TCCACGATACGTACGTTACCAACTG | K4 wzc sequencing |
| K5 R1 | TCAATAATGCGTACGTTACCTATTG | K5 wzc sequencing |
| K6 R1 | ACATTACCAACGGTACTAGCTTTAT | K6 wzc sequencing |
| K7 R1 | CTCACATTACCAATCGCACTTGATT | K7 wzc sequencing |
| K8 R1 | TCAATAATGCGAACATTCCCTATGG | K8 wzc sequencing |
| K9 R1 | TCAATAATTCTAACATTGCCAATAG | K9 wzc sequencing |
| K10 R1 | TCAATAATTCTAACATTACCAATCG | K10 wzc sequencing |
| K11 R1 | TCAATAATTCGAACGTTGCCAATTG | K11 wzc sequencing |
| K12 R1 | TCTACAATTCTGACATTACCAATAG | K12 wzc sequencing |
| K13 R1 | ATAATTCTGACATTACCTATTGCAC | K13 wzc sequencing |
| K14 R1 | GCATTATCAATGATTCTAACATTTCC | K14 wzc sequencing |
| K16 R1 | ACATTACCAATTGCACTTGATTTTG | K16 wzc sequencing |
| K17 R1 | TCAATGATTCTTACATTACCAATCG | K17 wzc sequencing |
| K18 R1 | TCAACAATCCGTACATTTCCAATTG | K18 wzc sequencing |
| K19 R1 | TCAATAATACGAACATTTCCAATAG | K19 wzc sequencing |
| K21 R1 | TCAATAACTCTTACATTTCCAATAG | K21 wzc sequencing |
| K22 R1 | GCTGTATCAATAATTCTAACATTAC | K22/K37 wzc sequencing |
| K23 R1 | ATTATACGCACATTACCAATCGCAC | K23 wzc sequencing |
| K24 R1 | ACATTCCCAATCGCGCTCGATTTTG | K24 wzc sequencing |
| K25 R1 | CCAATCGCGCTCGATTTAGCGATAT | K25 wzc sequencing |
| K26 R1 | ACATAACCAATAGCGCTTGATTTTG | K26 wzc sequencing |
| K27 R1 | TTGTCAATTATTCTGACATTACCTA | K27 wzc sequencing |
| K28 R1 | ATTGCACTAGACTTTGCAATATTCA | K28 wzc sequencing |
| K29 R1 | ATAATTCTTACATTTCCGATAGCAC | K29 wzc sequencing |
| K30 R1 | ATAGCAGAATCAACGATCCTAACAT | K30 wzc sequencing |
| K31 R1 | TCAATAATTCTCACATTACCAATTG | K31 wzc sequencing |
| K32 R1 | ATAATCCTAACATTACCAATGGCGC | K32 wzc sequencing |
| K33 R1 | GCATTATCAATAATCCTTACGTTTC | K33 wzc sequencing |
| K34 R1 | ATTCTAACGTTACCAATTGCACTTG | K34 wzc sequencing |
| K35 R1 | ATAATTCTTACGTTACCGATGGCAC | K35 wzc sequencing |
| K36 R1 | TCAATTATTCTCACATTACCAATAG | K36 wzc sequencing |
| K38 R1 | TCATCGATGATCCGTACATTTCCTA | K38 wzc sequencing |
| K39 R1 | ATAATCCTAACATTACCTATAGCAC | K39 wzc sequencing |
| K40 R1 | TCTATAATACGCACATTGCCAATGG | K40 wzc sequencing |
| K41 R1 | TCAATAATACGTACATTACCAATCG | K41 wzc sequencing |
| K42 R1 | CTCACATTACCAATCGCACTAGACT | K42 wzc sequencing |
| Primer | Sequences | Purpose |
| K43 R1 | TCAATAATTCTGACGTTTCCTATCG | K43 wzc sequencing |
| K44 R1 | TCGATAATTCTTACATTTCCAATTG | K44 wzc sequencing |
| K45 R1 | TCAATAATTCTAACATTGCCAATAG | K45 wzc sequencing |
| K46 R1 | ACATTTCCTATAGCACTCGATTTCG | K46 wzc sequencing |
| K47 R1 | TCAATAATTCTAACATTACCAATTG | K47 wzc sequencing |
| K48 R1 | ATAATTCTAACGTTCCCTATAGCAC | K48 wzc sequencing |
| K49 R1 | CTAACATTCCCAATTGCACTAGATT | K49 wzc sequencing |
| K51 R1 | ACGTTACCAATCGCACTAGACTTGG | K51 wzc sequencing |
| K52 R1 | TCAATAATCCGCACATTACCAATGG | K52 wzc sequencing |
| K53 R1 | ACGTTACCAATAGCGCTAGATTTTG | K53 wzc sequencing |
| K54 R1 | ATTGCACTTGATTTAGAAATTTCTAG | K54 wzc sequencing |
| K55 R1 | CTTACATTGCCAATAGCGCTGGATT | K55 wzc sequencing |
| K56 R1 | TCAATAATCCTAACATTACCTATCG | K56 wzc sequencing |
| K57 R1 | ACGTTACCTATAGCACTAGATTTGG | K57 wzc sequencing |
| K58 R1 | TTTCCAACTGTACTTGCTTTGGATA | K58 wzc sequencing |
| K59 R1 | TCAATTATGCGTACATTACCAATTG | K59 wzc sequencing |
| K60 R1 | ACATTACCTACCGCACTTGATTTTG | K60 wzc sequencing |
| K61 R1 | TCAATAATTCTAACGTTACCTATAG | K61 wzc sequencing |
| K63 R1 | TCTATAATTCGTACATTACCAATTG | K63 wzc sequencing |
| K64 R1 | GTAACGGCATTGTCGATAATTCTCA | K64 wzc sequencing |
| K65 R1 | TCAATTATACGTACATTACCGATCG | K65 wzc sequencing |
| K66 R1 | ACATTGCCAATTGCACTAGATTTTG | K66 wzc sequencing |
| K67 R1 | ACATTACCTATCGCACTAGATTTTG | K67 wzc sequencing |
| K68 R1 | TCGATAATTCGGACATTACCTATCG | K68 wzc sequencing |
| K69 R1 | TCTGCTATTGCTGAATCAACAATCC | K69 wzc sequencing |
| K70 R1 | TCAATTATCCTGACATTACCAATTG | K70 wzc sequencing |
| K71 R1 | CCAATTGCACTTGATTTGGCTATAT | K71 wzc sequencing |
| K72 R1 | TCAATTATTCTTACATTACCAACAG | K72 wzc sequencing |
| K74 R1 | TCTATAATCCGTACATTACCTATTG | K74 wzc sequencing |
| K79 R1 | TCAATAATACGCACATTACCAATAG | K79 wzc sequencing |
| K80 R1 | TCAATAATACGAACATTACCAATAG | K80 wzc sequencing |
| K81 R1 | TCAATTATGCGGACATTACCTATTG | K81 wzc sequencing |
| K82 R1 | TCGATAATACGAACATTGCCAATAG | K82 wzc sequencing |
| K4 R2 | CAGCAGATTGTTGATCGCCACCAAAC | K4 wzc sequencing |
| K5 R2 | TTGCAACTCAGTTATTGCTTGCAATT | K5 wzc sequencing |
| K6 R2 | AACAGTTTTTTGAATGGTCCATTTAG | K6 wzc sequencing |
| K7 R2 | GGTTATTGCTTTCAGACGCGTTAACT | K7 wzc sequencing |
| Primer | Sequences | Purpose |
| K8 R2 | CTGAAGAAGACTTATAGCTCTAAGTC | K8 wzc sequencing |
| K9 R2 | TCTTGTAAGTCAGTTATAGCTTGCAG | K9 wzc sequencing |
| K10 R2 | TTGTAAGTCTGAAATCGCTTTTAGTC | K10 wzc sequencing |
| K11 R2 | CTGTAATTCCGTAATAGCTTGCAAAC | K11 wzc sequencing |
| K12 R2 | TTGTAAATCGGTAATTGCTTTTAACT | K12 wzc sequencing |
| K13 R2 | TAGATTATTTATCGCCTTTAATTTTG | K13 wzc sequencing |
| K14 R2 | ATCTTGTAAATCAGAAATAGCTTTC | K14 wzc sequencing |
| K16 R2 | AATTGCTTTCAGTCTTGTAAAATATT | K16 wzc sequencing |
| K17 R2 | TTGTAATTTTGTAATTGCTTCGAGCC | K17 wzc sequencing |
| K18 R2 | TTGCAGATCTGTTATAGCTTTTAATT | K18 wzc sequencing |
| K19 R2 | TTGTAAGTCTGTTATTGCTTTTAAAC | K19 wzc sequencing |
| K21 R2 | CCTTAAATCATTAATAGCTTTTAGCT | K21 wzc sequencing |
| K22 R2 | ATTTTTCTGTAGTGAATCTAATACGT | K22/K37 wzc sequencing |
| K23 R2 | CAAATCGGTTATTGCTTGTAACTTTG | K23 wzc sequencing |
| K24 R2 | TATTGCTTTTAGACGGCTTAAATATT | K24 wzc sequencing |
| K25 R2 | TTTCAACTTGCTAATATACTCTATTT | K25 wzc sequencing |
| K26 R2 | TATTGCATTCAATCTTGTCAAGTATG | K26 wzc sequencing |
| K27 R2 | ATCCCGTAAATAGTTTATAGCATTTA | K27 wzc sequencing |
| K28 R2 | TAAATGGGGCATGTAAGTAACATTAT | K28 wzc sequencing |
| K29 R2 | CAAATCAGCTATAGCTTTTAATTTAC | K29 wzc sequencing |
| K30 R2 | GAATACTTTAAGTAAATTGTTAATGG | K30 wzc sequencing |
| K31 R2 | CTGTAAGTCCGTAATAGCTTTTAATC | K31 wzc sequencing |
| K32 R2 | CAAATCAGCTATTGCCTTTAATCGAC | K32 wzc sequencing |
| K33 R2 | GAATTTCTGTAGATTATTTATTGCTT | K33 wzc sequencing |
| K34 R2 | ATCACTAATTGCTTTCAGTTTTGGAA | K34 wzc sequencing |
| K35 R2 | CAAATTGTTTATTGCTTCAAGTTTTG | K35 wzc sequencing |
| K36 R2 | TTGTAAATCAGTGATAGCTTTCAGTT | K36 wzc sequencing |
| K38 R2 | ATCAAGTAAATCAGCTATAGCTTTCA | K38 wzc sequencing |
| K39 R2 | CAAGTCTGTTATCGCACGCAATTTTG | K39 wzc sequencing |
| K40 R2 | CTGCAAATCGGTAATAGCCTTTAGCT | K40 wzc sequencing |
| K41 R2 | TTGCAAGTCTTTAATTGCTCTCAGTT | K41 wzc sequencing |
| K42 R2 | TGAAATTGCTTTCAGTTTTGTTAACT | K42 wzc sequencing |
| K43 R2 | CTGCAGATCACTAATGGCTTTTAACT | K43 wzc sequencing |
| K44 R2 | TTGTAAATCAGTTATAGCTTTAAGCT | K44 wzc sequencing |
| K45 R2 | TTGTAAGTCAGTTATAGCTTGCAGCT | K45 wzc sequencing |
| K46 R2 | AATTGCTTTCAACTTATTAAGATAGG | K46 wzc sequencing |
| K47 R2 | CTGTAACGCACTTATTGCATCCAATT | K47 wzc sequencing |
| Primer | Sequences | Purpose |
| K48 R2 | AATATCAGATATCGCCTTTAACTTTG | K48 wzc sequencing |
| K49 R2 | ATTGATTGCTTTGAGTCTAGATAAGT | K49 wzc sequencing |
| K51 R2 | AATCGCTTTTAATCTTGTCTTATAAT | K51 wzc sequencing |
| K52 R2 | CTGAAGATCTGTTATAGCCTTCAATC | K52 wzc sequencing |
| K53 R2 | AATCGCTTTTAAACGTGATCTGTATA | K53 wzc sequencing |
| K54 R2 | TAATCTAGTCTTATATTTTATCTTAAA | K54 wzc sequencing |
| K55 R2 | ATTGATGGCCTCCAATTTTGAATAAT | K55 wzc sequencing |
| K56 R2 | TAAAAGATCGGTAATAGCCTGGAGCC | K56 wzc sequencing |
| K57 R2 | AATAGCTTTCAAACGACTTATATAGG | K57 wzc sequencing |
| K58 R2 | TGCTGTTACTTCTGCTATTTTCTTTA | K58 wzc sequencing |
| K59 R2 | TTGCAAATCAGTTATTGCCTTTAGAC | K59 wzc sequencing |
| K60 R2 | TATTGCTTTGAGCTGATCTACATAAT | K60 wzc sequencing |
| K61 R2 | CTGAAGATCATTAATAGCTTTTAACC | K61 wzc sequencing |
| K63 R2 | TTGTAAATTAGCTATCGCTTTATAGC | K63 wzc sequencing |
| K64 R2 | TGTTAATTGTTCTTGGATATCCGAAA | K64 wzc sequencing |
| K65 R2 | TTGCAAAGATGTTATAGCCTTTAATT | K65 wzc sequencing |
| K66 R2 | GATTGCTTTTAATGATGATACATAAG | K66 wzc sequencing |
| K67 R2 | AATTGCATTAAGTCGCGAAAGATATT | K67 wzc sequencing |
| K68 R2 | CTGCAGATTAGTTATAGCCTTCAGAC | K68 wzc sequencing |
| K69 R2 | AACACTTAACACTTTCTGAAGATTAT | K69 wzc sequencing |
| K70 R2 | TTGTAAATCAGTGATTGCTTTTAATT | K70 wzc sequencing |
| K71 R2 | ACTTAGTTTTGATATATATTTTATAT | K71 wzc sequencing |
| K72 R2 | CCTTAATTCATTAATTGCTTTTAACC | K72 wzc sequencing |
| K74 R2 | TTGTAGATCTGTGATCGCTTGCAAGC | K74 wzc sequencing |
| K79 R2 | CTGAAGGTCTGCTATAGCCTTCAATC | K79 wzc sequencing |
| K80 R2 | TTGCAGATCTGTAATTGCTTTCAAAC | K80 wzc sequencing |
| K81 R2 | TTGAAGATCCGTAATTGCTTTTAGGC | K81 wzc sequencing |
| K82 R2 | CTGTAAGTCTGTTATAGCTTTTAATT | K82 wzc sequencing |
